# Vaccine Hesitancy Phenomenon Evolution during Pregnancy over High-Risk Epidemiological Periods—“Repetitio Est Mater Studiorum”

**DOI:** 10.3390/vaccines11071207

**Published:** 2023-07-05

**Authors:** Valeria Herdea, Petruta Tarciuc, Raluca Ghionaru, Mircea Lupusoru, Elena Tataranu, Sergiu Chirila, Oana Rosu, Cristina Oana Marginean, Eugene Leibovitz, Smaranda Diaconescu

**Affiliations:** 1 Doctoral School, “George Emil Palade” University of Medicine, Pharmacy, Science, and Technology of Targu Mures, 540142 Targu Mures, Romania; 2 Romanian Association for Pediatric Education in Family Medicine, 021507 Bucharest, Romania; 3 Department of Physiology, Carol Davila University of Medicine and Pharmacy, 020021 Bucharest, Romania; 4 Faculty of Medicine and Biological Sciences, “Stefan cel Mare” University of Suceava, 720229 Suceava, Romania; 5 Faculty of Medicine, Ovidius University of Constanta, 900470 Constanta, Romania; 6 Department of Pediatrics, “George Emil Palade” University of Medicine, Pharmacy, Science, andTechnology of Targu Mures, 540142 Targu Mures, Romania; marginean.oana@gmail.com; 7 The Pediatric Infectious Disease Unit, Soroka University Medical Center, Beer-Sheva 85025, Israel; 8 Faculty of Health Sciences, Ben-Gurion University of the Negev, Beer-Sheva 85025, Israel; 9 Medical-Surgical Department, Faculty of Medicine, Titu Maiorescu University of Medicine and Pharmacy, 031593 Bucharest, Romania; turti23@yahoo.com

**Keywords:** vaccination acceptance, vaccination hesitancy, pregnant woman, measles, pertussis vaccine, COVID-19, preventive healthcare, education

## Abstract

(1) Background: The recent epidemiological events were high-stress level generators for humanity, particularly for pregnant women, influencing their attitude, behavior, and decisions regarding vaccination during pregnancy or regarding their future child. The aim of this study was to analyze the anti-pertussis vaccination decision-shaping factors in pregnant women during two epidemiological periods: the measles epidemic and the COVID-19 pandemic. (2) Methods: Two groups of pregnant women were invited to be part of a medical education program, having as the main theme the infectious disease risks and their prevention through vaccination. Before launching the program, participants received a 12-item questionnaire. From a total number of 362 pregnant women enrolled in the study, 182 participated in 2019, and 180 participated in 2022. (3) Results: The socio-demographic data revealed that the age of pregnant women participating in medical education programs increased in 2022 by 1.7 years (*p* < 0.01). In vitro fertilization was reported in a significantly higher proportion (20% in 2022 vs 9.8% in 2019, *p* < 0.01). Participation in community-initiated educational programs almost doubled during the pandemic time from 18.7% in 2019 to 33.9% in 2022 (*p* < 0.01). Pertussis vaccine acceptancy (VA) dropped from 85% in 2019 to 44.4% in 2022 (*p* < 0.01) (4) Conclusions: In this study, we reported fast-growing vaccine hesitancy and severe declared vaccine reluctance. The results of this complex long-term study, which evaluated pregnant women over several years, showed a five-fold increase in the percentage of pregnant women who disagreed with personal pertussis vaccination. This draws attention to the risks of pertussis epidemic outbreaks in pregnant women and their future infants in the first couple of months of life before the initiation of vaccination.

## 1. Introduction

The last seven years were marked by the measles epidemic (2017–2019) and the COVID-19 pandemic (2020–2023), two severe epidemiological periods that affected the entire world. These events were high-stress level generators worldwide [1]. A special category was represented by pregnant women in which stress could negatively influence their attitudes and behavior, thus influencing their decisions regarding vaccination during pregnancy [2,3,4,5,6,7,8].

According to the World Health Organization, measles cases reached 869,770 in 2019, with over 200,705 lives being lost worldwide [9]. Across Europe, measles cases tripled during 2017–2018 [9]; in total, 28% of European children below 5 years old were affected by measles. From the beginning of the measles epidemic in Romania until late 2019, 18,578 measles cases were registered, with 10,152 (54.6%) being diagnosed in children under 4 years old. A total number of 64 deaths among children was recorded during this period (2016–2019), most of these cases being diagnosed in unvaccinated children [10]. At the European level, during the same time frame (2016–2019), there were 93 deaths recorded attributed to measles infections [11].

Starting on 16 March 2020, Romania was affected by the COVID-19 pandemic. Until 1 April 2023, almost 700 million cases of COVID-19 and almost 7 million deaths were recorded worldwide; until this date, Romania reported 3,367,921 COVID-19 cases and 67,858 deaths [12].

In the last decades of modern history, researchers have struggled to control severe infectious diseases through vaccination [13]. Every year, 1.5 million children die because they do not have access to vaccines. Between 2010 and 2020, it is estimated that vaccines and vaccination saved 25 million lives, representing “five lives saved per minute” globally [14]. Despite the recorded results, in the last two decades, increasing opposition to vaccines and vaccination has been reported. Vaccine hesitancy, defined as “a state of indecisiveness regarding a vaccination decision” [15,16], and vaccine refusal, defined as the “result of ever-changing experience and continual risk assessment” [17], are among the top 10 threats to global health according to World Health Organization [18]. 

According to new scientific research data, including a 2020 large meta-analysis, with references from 120 studies performed in 30 countries, there is a globally low coverage for influenza and pertussis vaccination during the sensitive period of pregnancy, ranging from 0% to 70%. The missed vaccinations for pregnant women represent lost opportunities for preserving the health of future mothers and infants too. An interesting finding referred to the role of physicians in the counseling and medical education of pregnant women. Accordingly, the healthcare professionals’ recommendations could raise the vaccine acceptance rate during pregnancy 10–12 times [19,20,21].

In Romania, the general vaccination coverage for the whole population was affected during the later years, following the global trend of increasing vaccine hesitancy. When comparing the vaccine coverage during the first year of the COVID-19 pandemic (2020) with the vaccination coverage during the 2019 measles epidemic, a significant decline was observed for all vaccines included in the Romanian National Immunization Program. According to a recent study comparing the general vaccine coverage in children younger than 1 year of age from January 2019 to June 2019 (the measles epidemic) and January 2020 to June 2020 (the COVID-19 pandemic), the measles–mumps–rubella vaccine coverage for administration at the age of 12 months was 64.29% in 2019 vs 55.88% in 2020, and for the diphtheria–tetanus–pertussis vaccine, it was 71.59% in 2019 vs 62.08% in 2020 [22]. 

Another modern threat is a new type of epidemic–pandemic—the infodemic, referring to an abundance of fake news that shapes and changes an important percentage of the people’s opinion on disease prevention through immunization [23]. Recent publications, which focus on communication and public relations, showed that the fake news phenomenon, described as a new type of “arms race” of the 21st century, will change Generation Omega’s way of life, their attitude, and their essential decision-making processes during their teenage years and that it will also change the communication and social interaction skills of future adults [24,25]. Approximately 80% of all internet users search for health information. Considering the unregulated environment, misinformation can easily be transmitted. This has the potential to lead, in the end, to negative results, behavior changes, misjudgment, the spread of doubt, and fake news on internet channels, for parents and even for a part of healthcare personnel who are not directly involved in immunization activities [26,27]. An important step to lower parental vaccine hesitancy resides in the training of the medical human resources involved in prevention, especially in the communication of the protective role of vaccination. Students and residents remain the first groups in need of training for optimal patient-centered communication starting from universities during residency and oriented towards understanding patients’ needs and expectations and towards understanding the risk of infectious diseases and the protective role of the vaccination act [28]. This type of education could help young doctors reduce their possible hesitancy regarding infectious disease prevention through new vaccines or new innovative techniques of vaccine production [29], a process similar to that which occurred during the COVID-19 pandemic.

The purpose of the present study is to analyze (1) the shaping factors of the decision-making process of anti-pertussis vaccination in pregnant women and (2) to analyze the changes in the vaccine hesitancy phenomenon in pregnant women in 2019–2022 during two challenging epidemiological periods: the measles epidemic and the COVID-19 pandemic.

## 2. Materials and Methods

Three years apart, in two different epidemiological circumstances, 2019 (measles epidemic) and 2022 (COVID-19 pandemic), two groups of pregnant women from a metropolitan area in the southeast of Romania, the city of Bucharest (Romania’s capital), were invited to be part of a medical education program. 

One of the objectives of this program was to analyze pregnant women’s attitudes regarding their personal and their offspring’s vaccination and development. The program was developed by research team members of the Romanian Association for Pediatric Education in Family Medicine, a professional organization of family doctors. The design of the program, done by primary healthcare practitioners, was based on the educational needs of the parents in the first years of their children’s lives and was focused on the prevention of infectious diseases, vaccination, nutrition, and neuropsychological development of the child. The participants were introduced to the education program through social media channels in both periods. Before the initiation of the program, the participants received and completed a 12-item questionnaire structured to evaluate socio-demographic data, pregnancy follow-up, infectious disease risk perception gaps, interest in participating in medical education programs during pregnancy, agreed topics, and personal attitudes regarding pertussis vaccine acceptance during pregnancy.

The questionnaire presented a short introduction to the main purpose of the medical education program dedicated to pregnant women (Supplementary Files S1 and S2). Participants were informed regarding data protection and Medical Academy Ethics Committee approval. The questions were structured to analyze socio-demographic data (age, living environment, and educational level), pregnancy follow-up (gestational age, number of offspring, and conceiving method), interest in participating in medical education programs during pregnancy (including preferred forms of education), infectious disease risk perception gaps and preferred topics, personal attitudes regarding pertussis vaccine acceptance (VA) during pregnancy, and agreement/disagreement to receive informative materials connected to the main theme of the present medical education program.

To facilitate participants’ access, the questionnaires were available online for 6 weeks in 2019 and another 6 weeks in 2022. The survey complied with Romanian legislation (Law 190/2018) and GDPR—the General Data Protection Regulation 679/2016. The Ethics Committee of the Academy of Medical Sciences, National Bioethics Commission of Medicines and Medical Devices, approved the study under number 1SNi/21.02.2019, and Romanian Association of Pediatric Education in Family Medicine (AREPMF) approved the study under approval no 3/19/2020 from 19 March 2020. Questionnaire completion represented the agreement of the pregnant women to be enrolled in the study. The pregnant women were informed that the questionnaire was part of a doctoral study, that the results would be published in a scientific medical journal, and that all participants would have free access to the results of the study on request. There was no incentive for completing the questionnaire nor any restrictions in case of not answering the questions. Participation in the educational program was free of charge.

This entire process took place as a larger education program targeting pregnant women implemented between 2018 and 2022. The research conducted within this project had several main interconnected aims: evaluation of vaccine acceptance among pregnant women and evaluation of the anti-pertussis immune status of pregnant women [27]; evaluation of vaccination coverage for children aged 0–1 year old in Romania, with focus on the specific epidemiological context [13]; and the aim of the current study, the shaping factors of the decision-making process of anti-pertussis vaccination in pregnant women during this time frame and changes in vaccine acceptance.

From a total number of 352 pregnant women who were invited in 2019 to participate in the medical educational program, 182 chose to complete the questionnaire. For the year 2022, a number of 346 pregnant women were invited to participate in the medical educational program, and a number of 180 agreed to answer the questionnaire.

A statistical analysis was conducted using IBM SPSS Statistics version 28. Descriptive statistics were used for characterizing the variables (mean, standard deviation, median for continuous variables, and proportions for nominal values). The chi-square test was used for testing associations, while for testing differences between numerical continuous data, we used a *t*-test when comparing the results from 2019 with the results from 2022. All variables were tested for normality using visual methods and the Shapiro–Wilk test, and we decided that their distribution did not differ from a normal distribution for any of the analyzed variables. To control for independent variables within the samples, we ran a multinomial logistic regression test. The decision to use this test was driven by the fact that the dependent variable (attitude regarding anti-pertussis vaccination during pregnancy) had three possible answers: “Agree”, “Mostly agree”, and “Disagree”. We considered that the results were statistically significant if a *p*-value less than 0.05 was obtained.

## 3. Results

### 3.1. Socio-Demographic Data

The present study evaluated the opinions of 362 pregnant women: in total, 182 pregnant women completed the questionnaire in 2019, and 180 pregnant women completed it in 2022.

**Figure 1:**
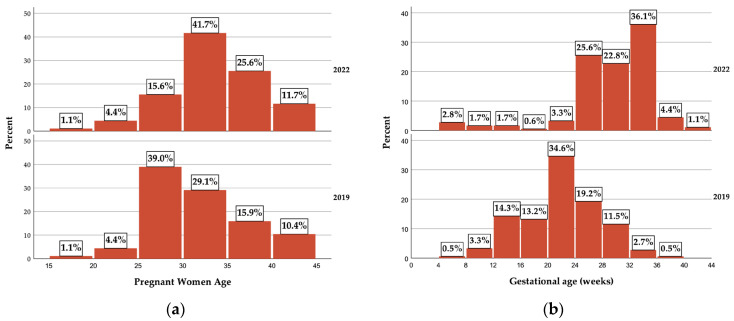
(**a**) Pregnant woman distribution according to age and (**b**) pregnant woman distribution according to gestational age.

Table 1 and Figure 1a summarize the socio-demographic aspects related to the age, environment, and educational level of the pregnant women. The average age of the pregnant women was higher for the 2022 sample, with a mean difference of 1.72 years (CI 95% 0.68–2.75 years, *p* < 0.01). The main difference, based on age group, was observed for the 25 to 30 year old pregnant women, which, in 2019, represented almost 40%, while, in 2022, they represented approximately 16%.

There was a statistically significantly higher proportion of pregnant women that reported their educational level as being “high school” in 2022 (22.2%) compared with the sample from 2019 (3.8%). The percentage of university-graduated pregnant women in 2019 reached 96.2% compared with that of 72.8% in 2022.

### 3.2. Pregnancy Follow-Up

The overall gestational age, measured in weeks, was higher for the 2022 pregnant woman sample, with a mean difference of 7.22 weeks (95% CI 5.92–8.5 weeks, *p* < 0.01) (Figure 1b). In 2022, the sample presented a higher proportion of pregnant women with a higher pregnancy age, 90% being in the third trimester, while, for the 2019 sample, most of them were in the second trimester (approximately 81%). 

The differences in the proportion of pregnant women that used in vitro fertilization or other means (different from natural conception) for conception were recorded: in total, it was 36/180 (20%) in 2022 compared with 18/182 (9.8%) in 2019 (Table 2).

### 3.3. Educational Objectives

**Figure 2:**
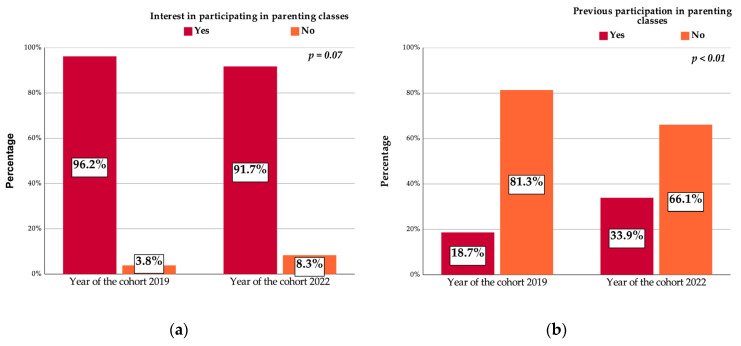
(**a**) Interest in participating in parenting classes and (**b**) previous participation in parenting classes.

More than 96% of the pregnant women from the 2019 sample and 91.7% from the 2022 sample expressed their interest to participate in parenting programs (*p* = 0.07, Figure 2a). At the same time, the percentage of pregnant women that previously took part in parenting programs raised during the pandemic time, going up from 18.7% in 2019 to 33.9% in 2022 (*p* < 0.01) (Figure 2). Figure 2a depicts the intention to participate in parenting classes, which was significantly higher when compared to the data from Figure 2b, where the actual previous participation in other educational programs was confirmed by the pregnant women.

On the four educational topics proposed to the pregnant women, differences in preferences were not different in a statistically significant manner between 2019 and 2022 (Table 3), except for a decrease in the interest of enrolled pregnant women regarding infectious disease prevention (*p* < 0.01), where a 15% decrease in the interest in this topic was observed.

### 3.4. Personal Attitudes Regarding Pertussis Vaccine Acceptance during Pregnancy

**Figure 3:**
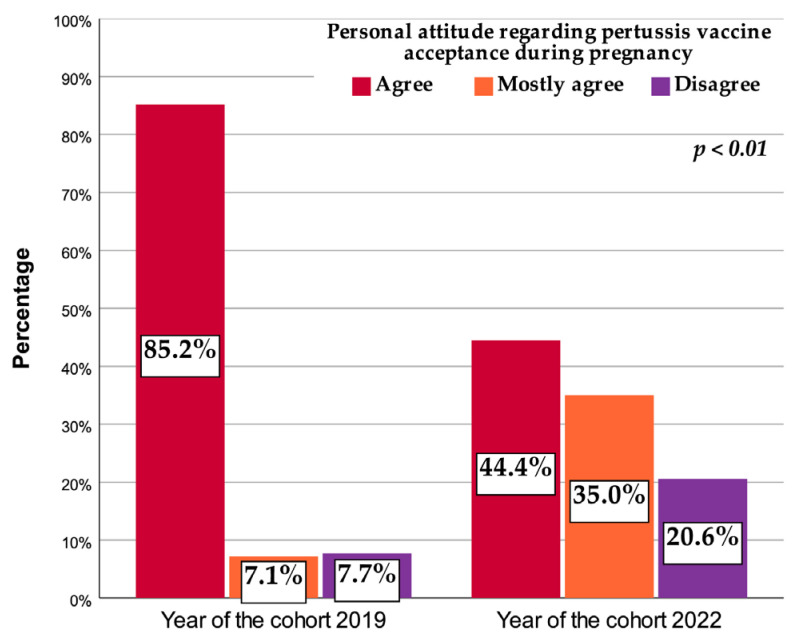
Personal attitude regarding pertussis vaccine acceptance during pregnancy.

The percentage of pregnant women that would readily accept pertussis vaccination decreased in 2022 compared to 2019 from 85.2% to 44.4% (Figure 3). A five-fold increase was observed in the percentage of pregnant women hesitant towards pertussis vaccination during pregnancy (from 7.1% in 2019 to 35% in 2022) together with an almost three-fold increase in the percentage of pregnant women disagreeing with pertussis vaccination (from 7.7% in 2019 to 20.6% in 2022). 

We ran a multinomial logistic regression analysis to determine the role of the time difference and the demographic and socio-economic characteristics of the pregnant women in two of their attitudes regarding pertussis vaccine acceptance during pregnancy (Table 4). The model included the following variables: the year of the cohort, educational level, and environment as nominal independent variables and the pregnant women’s age as a continuous independent variable. We considered the “Agree” status to be the reference category, and we compared the “Mostly agree” category and the “Disagree” category to the “Agree” category.

Comparing the “Agree” dependent category with the “Mostly agree” dependent category, we observed that two independent variables influenced the decision in a statistically significant way: the year of the cohort, the pregnant women from the 2019 cohort being less likely to “Mostly agree” with anti-pertussis vaccination during pregnancy (*p* < 0.01), and the educational level, where pregnant women with a lower educational level had higher odds of choosing “Mostly agree”.

Comparing the “Agree” dependent category with the “Disagree” dependent category, we observed that the pregnant women’s age and the year of the cohort had statistically significant results. Thus, as the age of the pregnant women increased, there were higher odds of disagreeing with anti-pertussis vaccination during pregnancy, and, also, the year of the cohort influenced the attitude towards anti-pertussis vaccination during pregnancy in a statistically significant way, the pre-pandemic year being associated with significantly lower odds of refusing vaccination.

## 4. Discussion

In the present study, we evaluated how pregnant women’s vaccine acceptance patterns for vaccination in general and, particularly, concerning anti-pertussis vaccination changed during the measles epidemic and the COVID-19 pandemic.

The pregnant women from both periods participated in medical educational programs aimed at informing people about the risk of a pertussis epidemic outbreak not just for unvaccinated pregnant women but also for their future infants, at least in the first couple of months of life until vaccinated according to the National Vaccination Program.

According to the United Nations’ data, 529,000 women die worldwide every year during childbirth, and 300 million suffer from complications during pregnancy. The best solutions to minimize the occurrence of these preventable deaths remain to be access to proper medical services and early health education for girls and young pregnant women [30]. The World Health Organization 2023 global initiative “Health for All” promotes, among other aspects, the same solutions [31]. A recent study, that took place between 2020 and 2022 in Bucharest (a metropolitan South-East area of Romania) and had as one of its main purposes the analysis of protective antibodies’ levels against pertussis among a cohort of pregnant women, showed a concerning waning humoral immunity against *Bordetella pertussis* infections in 91.9% of the pregnant women [32]. 

From 2016 to 2019, during the measles epidemic period, public communication efforts were targeted at patients’ needs, with a focus on risk groups [6,9,33]. In the United States, during the measles epidemic, public health responses included, among other measures, communication campaigns, the delivery of printed materials, and media releases. All these methods were used as a means of providing information on the disease and prevention through vaccination, which was adapted to the communities under high risk [34]. All successful education programs were also based and structured according to the main needs that were identified for pregnant women [35].

The recent history of infectious disease outbreaks exposed pregnant women to supplementary risks. Medical education programs were conducted mainly online during the COVID-19 pandemic and were adapted to the specific conditions of stress and isolation. At the beginning of the pandemic, the whole population, including pregnant women, was confronted with a new, unknown disease. The lack of clear evidence-based medical information, the lack of protective supplies and medical supplies, and incertitude negatively influenced the population and also medical staff behavior [36]. After a few months, online education became ubiquitous, and people largely adopted the new teaching and learning methods [1,2,3,4,5]. As a result, access to training programs was improved. This was also observed in our present study, which showed that the percentage of pregnant women taking part in parenting programs doubled (compared with 2019) during the COVID-19 pandemic. 

In addition, we showed in this study that pregnant women were more interested in accessing an education program in the first two trimesters of their pregnancy. For the 2022 cohort, even though the average age and gestational age were significantly higher compared with the 2019 cohort, approximately one in three pregnant women reported previous participation in specially designed educational programs. An important aspect to be mentioned, apart from “quantity”, refers to the quality of the programs that pregnant women attended. If, before the pandemic, most of the courses were frontal, thus facilitating interaction, the shift to online education brought important changes in the way participants interacted amongst themselves and with the teaching staff, adding the possibility of losing attention due to distractions and providing, sometimes, an overwhelming quantity of information [37]. Interestingly, despite the higher grade of previous participation in multiple educational programs dedicated to pregnant women, an important decrease in vaccine acceptance was reported in our study, leading to the possibility that, during the later years, other factors were more important in shaping the opinion of pregnant women with respect to vaccine acceptance than the education provided by trained medical staff [19,20,38].

The decision-making process is a multi-factorial one, so accessing medical educational programs in our study was correlated with the pregnant women having an older age and a higher educational level, as most of the participants had graduated from university-level studies (more than 70% of the total sample, with a higher percentage in the sample from 2022).

The pregnant women attending parenting classes in 2022 were older than the pregnant women who participated in the 2019 classes. This can be considered as an indication of the fact that pregnancy was achieved at an older age in the pandemic period. The results fall under the general country-level statistics, which show that the average age of pregnant women increased in 2022, with a mean value of 2.4 years (from 27 years old in 2019 to 29.4 years old in 2022) [39,40,41]. 

While the age of the pregnant women increased, their level of education was significantly lower in the 2022 cohort compared to the 2019 one. A higher proportion of pregnant women reported their educational level as being at the “high school” level in 2022 (22.2%) compared with the sample from 2019 (3.8%). We noticed that, for the 2022 sample, the pregnant women presented a tendency towards being older with a lower educational level. These socio-demographic characteristics combined seem to favor vaccine mistrust, an aspect observed in other recently conducted studies [42]. We conducted multinomial logistic regression to control for these factors (age and level of education), together with the living environment and the cohort they were part of (2019 or 2022), to identify the independent role each of these factors had in shaping their anti-pertussis vaccination decision. We observed that the change from the status of “Agree” to “Mostly agree” was influenced in a statistically significant way by the year of the cohort (pregnant women in 2022 having the odds of being more reluctant) but also by the level of education (a lower level of education increased the odds of being more reluctant to vaccination). When comparing the difference between vaccine acceptance and vaccine refusal, we observed that the year of the cohort had a statistically significant influence (odds of refusing vaccination were higher for the 2022 cohort) but that the age of the pregnant women also did (older women had higher odds of refusing anti-pertussis vaccination). We thus observed that the epidemic period and the pandemic time had an independent, statistically significant role in lowering the anti-pertussis vaccination acceptance rate during pregnancy, influencing both vaccine hesitancy and vaccine refusal. 

As the main topic of the educational program was to inform pregnant women on different aspects related to their health and the health of the future newborn, these offered great support in shaping their decision. The instructional programs most probably positively shaped the pregnant women’s prevention decision-making process related to their vaccination and the vaccination of their offspring as it was demonstrated in previous studies [32]. 

Vaccination decision making is a long-term process. During pregnancy, pregnant women could be influenced by many factors (infodemic, epidemic, pandemic, rumors, and fake news), thus the gestational age can play an important role as a decision-making factor. Being aware of the pregnancy, the women increased their awareness of the existing information, and, considering the period in which the unfiltered information was readily available, the possibility of increasing their doubts related to vaccination in general and pertussis vaccination in particular was very high. Reports show that pregnant women with a higher gestational age expressed lower COVID-19 vaccination acceptance [43].

Another interesting point of view revealed during the study was that the number of pregnancies obtained through in vitro fertilization increased significantly during the COVID-19 pandemic. Existing data support the idea that, after successful IVF, couples experienced more psychological problems during pregnancy [44], especially anxiety and stress, caused mainly by concern for the well-being of the unborn child. Studies conducted during the COVID-19 pandemic identified IVF as being positively associated with vaccine hesitancy [45]. 

We reported a change in the preferred education methods from the measles epidemic to the SARS-CoV-2 pandemic from frontal workshops in 2019 to online classes in 2022.

According to Hirschberg et al., communication and patient awareness during the 2016–2018 USA measles outbreak were public health policy principles. By applying them, the vaccine acceptance rate increased [46]. A huge quantity of information in various forms was available online during the COVID-19 pandemic [2,3,4,5,34,47,48]. Romania faced the same challenges for both the measles [48,49] and the COVID-19 pandemic [7,8,25]. In the present study we noticed that, despite the increase in interest in participation in educational programs stated by pregnant women from 2019, the actual participation in such programs was lower compared to the 2022 cohort. The most reasonable explanation for this finding is related to the change in the way that the educational programs were conducted. Before the COVID-19 pandemic, most of the educational programs were conducted frontally, while, following the pandemic occurrence, most of the educational programs dedicated to pregnant women used the online environment. This change, together with the restrictions imposed on the population, facilitated online participation.

While there is a major worldwide interest in vaccine safety and efficacy, we could see, in the present study, a degree of saturation for discussing and learning infectious disease themes that were most probably generated by the informational “avalanche” during the latter years.

During high-risk epidemiological circumstances, pregnant women were confronted with information of different qualities, some medical-based evidence but some fake news. This information addressed the stress and fear levels and may have affected pregnancy evolution and future infant safety, adding general mistrust and overall insecurity. Finally, all these shaping factors generated new attitudes and decisions, which translated into general vaccine hesitancy and reluctance [1,2,3,4,5,6,7,8,23,24,25,35,50,51,52]. 

Our results showed a concerning vaccine hesitancy phenomenon during the COVID-19 pandemic, with a significant decrease in anti-pertussis vaccine acceptance. Vaccine hesitancy was defined as a “crisis of trust” rather than a “war on science” [53]. If vaccine hesitancy, in general, during the measles epidemic was generated by combined factors (an insufficient vaccine supply, vaccine reluctance, and sub-optimal communication addressed to high-risk clusters), vaccine hesitancy during the COVID-19 pandemic was mainly caused by the infodemic [24,25] and also by the inconsistency of pandemic preparedness and its management [1,3,4,5].

The last decade saw an increase in alcohol [54], tobacco [55], and drug [56] addiction among pregnant women. Addictions severely influence the pregnant woman and the future child’s health, influencing the pregnant woman’s ability to make the right decisions for her and the future child. Each of these products can expose both the pregnant woman and the future child to severe disorders of the immune system, in utero death, premature birth, malformations, and severe immune deficits, generating huge social costs and efforts for mothers’ and infants’ recovery. Mother–child interdependence remains a great challenge, especially in managing the presented situations. Early preventive medical education for pregnant women could be one of the solutions that could reduce the risks associated with addictions. 

Vaccine acceptance’s strongest predictors are considered to be pregnant women’s confidence in vaccine safety and efficacy, trust in the healthcare system, concerns about the risk of infectious diseases, and participation in appropriate education campaigns [47,57].

Vaccine reluctance’s predictors are considered to be the pregnant women’s fear of side effects, the lack of medical education programs elaborated by professionals in the medical field, the fake news/infodemic during the pandemic, anti-vaccination campaigns, and the lack of protective legislation for potential side effects after vaccinations [32,58,59].

Our study has some limitations related to the relatively small group size enrolled, its completion in a small and defined geographical area, and the possible enrolment of a select population concerning its higher educational level. While the purpose of the current study was to evaluate the way in which anti-pertussis vaccine acceptance was influenced by the major epidemiological events that affected the population in the recent years, more studies are needed to fully understand how general vaccine acceptance was influenced and also to better understand the shaping factors of vaccine acceptance and the determinants for vaccine hesitancy and vaccine reluctance in pregnant women.

## 5. Conclusions

Our data reached some concerning conclusions with respect to the vaccine acceptance of pregnant women, reporting that, in parallel to an increase in the degree of severity of the epidemiological context, anti-pertussis vaccine acceptancy during pregnancy decreased. We showed fast-growing anti-pertussis vaccine hesitancy (a five-fold increase was observed in the percentage of pregnant women hesitant towards pertussis vaccination during pregnancy) and severe declared vaccine reluctance (an almost three-fold increase in the percentage of pregnant women disagreeing with pertussis vaccination). The results of this study draw attention to the risks of a pertussis epidemic outbreak in pregnant women and future infants in the first months of life.

## Figures and Tables

**Table 1:** Socio-demographic characteristics.

Category	Total (n = 362)	2019 (n = 182)	2022 (n = 180)	*p* Value
Age (years)				
Mean ± SD	32.2 ± 5.05	31.3 ± 5.2	33.1 ± 4.8	<0.01
Median	32	30.5	33	
Environment				
Urban	346 (95.6%)	176 (96.7%)	170 (94.4%)	0.29
Rural	16 (4.4%)	6 (3.3%)	10 (5.6%)	
Educational level				
Elementary School	9 (2.5%)	0 (0%)	9 (5%)	<0.01
High School	47 (13%)	7 (3.8%)	40 (22.2%)	
University	306 (84.5%)	175 (96.2%)	131 (72.8%)	

**Table 2:** Pregnancy follow-up aspects.

Category	Total (n = 362)	2019 (n = 182)	2022 (n = 180)	*p* Value
Gestational age (weeks)				
Mean ± SD	24.9 ± 7.2	21.4 ± 5.9	28.6 ± 6.6	<0.01
Median	25	21	29	
Pregnancy Type				
Single Pregnancy	302 (83.7%)	152 (84%)	150 (83.3%)	0.87
Multiple Pregnancy	59 (16.3%)	29 (16%)	30 (16.7%)	
Conception Type				
Natural Conception	301 (83.1%)	164 (90%)	137 (76.1%)	<0.01
In Vitro Fertilization	45 (12.4%)	17 (9.3%)	28 (15.6%)	
Other Means	9 (2.5%)	1 (0.5%)	8 (4.4%)	
No answer	7 (1.9%)	0 (0%)	7 (3.9%)	

**Table 3:** Agreed topic distribution.

Category	Total (n = 362)	2019 (n = 182)	2022 (n = 180)	*p* Value
Breastfeeding/0–12 month infant nutrition				
Yes	321 (88.7%)	167 (91.8%)	154 (85.6%)	0.63
No	41 (11.3%)	15 (8.2%)	26 (14.4%)	
0–12 month child’s neuropsychic development				
Yes	306 (84.5%)	156 (85.7%)	150 (83.3%)	0.53
No	56 (15.5%)	26 (14.3%)	30 (16.7%)	
Infectious disease prevention				
Yes	286 (79%)	158 (86.8%)	128 (71.1%)	<0.01
No	76 (21%)	24 (13.2%)	52 (28.9%)	
Immunization				
Yes	292 (80.7%)	149 (81.9%)	143 (79.4%)	0.56
No	70 (19.3%)	33 (18.1%)	37 (20.6%)	

**Table 4:** Attitude towards personal vaccination during pregnancy.

Attitude towards Personal Vaccination during Pregnancy ^a^		B	Std. Error	Wald	df	Sig.	Exp(B)	95% Confidence Interval for Exp(B)	
								Lower Bound	Upper Bound
Mostly agree	Intercept	−17.999	1.045	296.67	1	0.00			
	Pregnant Women’s Age	0.012	0.031	0.156	1	0.69	1.012	0.953	1.076
	(Year of the cohort = 2019)	−2.170	0.355	37.28	1	0.00	0.114	0.057	0.229
	(Year of the cohort = 2022)	0 ^b^	.	.	0	.	.	.	.
	(Educational Level = Elementary School)	2.223	1.091	4.15	1	0.04	9.239	1.088	78.471
	(Educational Level = High School)	0.075	0.390	0.037	1	0.85	1.078	0.502	2.315
	(Educational Level = University)	0 ^b^	.	.	0	.	.	.	.
	(Environment = Urban)	17.343	0.000	.	1	.	34,028,683.3	34,028,683.3	34,028,683.3
	(Environment = Rural)	0 ^b^	.	.	0	.	.	.	.
Disagree	Intercept	−4.312	1.421	9.21	1	0.002			
	Pregnant Women’s Age	0.090	0.035	6.736	1	0.009	1.094	1.022	1.170
	(Year of the cohort = 2019)	−1.707	0.361	22.32	1	0.000	0.181	0.089	0.368
	(Year of the cohort = 2022)	0 ^b^	.	.	0	.	.	.	.
	(Educational Level = Elementary School)	−16.730	5213.72	0.000	1	0.99	5.424E−8	0.000	-.^c^
	(Educational Level = High School)	−0.764	0.547	1.952	1	0.16	0.466	0.159	1.360
	(Educational Level = University)	0 ^b^	.	.	0	.	.	.	.
	(Environment = Urban)	0.745	0.825	0.815	1	0.36	2.106	0.418	10.610
	(Environment = Rural)	0 ^b^	.	.	0	.	.	.	.

^a^ The reference category is Agree. ^b^ This parameter was set to zero because it is redundant. ^c^ Floating point overflow occurred while computing this statistic. Its value was therefore set to system missing.

## Data Availability

The datasets used and/or analyzed during the current study are available from the corresponding author upon reasonable request.

## Supplementary Materials

The following supporting information can be downloaded at https://www.mdpi.com/article/10.3390/vaccines11071207/s1, File S1: Questionnaire addressed to pregnant women and File S2: Study flow.
