# Supplementary material for: Vaccine Hesitancy Phenomenon Evolution during Pregnancy over High-Risk Epidemiological Periods—“Repetitio Est Mater Studiorum”
Source: Vaccines (Basel). 2023 Jul 5;11(7):1207. doi: 10.3390/vaccines11071207 (PMC10384756; doi:10.3390/vaccines11071207)
Supplement: Supplementary file 1 [file vaccines-11-01207-s001.zip › S1 Questionnaire addressed to pregnant women.pdf]

Questionnaire addressed to pregnant women

We invite you to take 2 minutes of your time to complete the bellow questionnaire.

Why to complete?

Accurate medical information enables childbearing ages women to take an informed decision regarding self protection against infectious diseases during pregnancy and for the future infant during the first couple months until she/he will be immunized according the National Vaccination Program.

Where will be used collected data?

The questionnaire is intended for a doctoral research study. The results will be published in a scientific medical journal. Participants will have free access to the results of the study by request.

Data protection.

The data collected in this questionnaire remain anonymous. Completion of the questionnaire is not mandatory. Withdrawal from the study at any time without any explanation is a personal option. Participation in the medical education program is free of charge. Completing the questionnaire represents your agreement to participate. The questionnaire completion signified your agreement to enter into the study. The survey complied with the Romanian legislation (Law 190/2018) and GDPR – the General Data Protection Regulation 679/2016. The study was approved by the Ethics Committee of the Academy of Medical Sciences, National Bioethics Commission of Medicines and Medical Devices, under the number 1SNi/21.02.2019.

Thank you!

1. Age (years)

2. Residence (urban/rural)

3. Educational level (primary, secondary, high-school, university, post-university studies)

4. Gestational age (weeks)

5. Pregnancy type (singleton pregnancy, twin pregnancy, > two fetuses)

6. Pregnancy (natural [physiological], assisted reproduction technology (ART) IVF, other means of medically assisted human reproduction

7. Participation during pregnancy in community-initiated educational parenting programs courses designed to help in increasing your knowledge and understanding in parenting:

a. Yes

b. No

8. Would you be interested in participating in such a course during present pregnancy?

a. Yes

b. No

9. What would be your preferred forms of education?

a. Frontal: Discussions with the medical staff (doctor/nurse)/ lecturer/workshops/conferences/classes

b. On-line: Webinar/E-book

c. Printed materials: brochures, books, printed flyers

d. Electronic messaging (e- mail, what’s up, messages)

10. What would be your preferred themes for improving your parental knowledge/undestanding?

a. breastfeeding/ nutrition of the infant aged 0-12 months,

b. neuropsychological development of the child

c. infectious diseases prevention

d. vaccination

11. Vaccination of pregnant women during the period of 27-36 weeks of pregnancy allows to protect the pregnant woman from pertussis infection (whooping cough) and it allows to protect the future child in the first couple of months of life, up to the moment when she/ he will be vaccinated according to the National Vaccination Program. What is your opinion related to personal anti- pertussis vaccination during pregnancy?

a. Agreed

b. Mostly agree

c. I do not agree

12. Do you agree to receive informative materials by e-mail connected to the main theme of this medical education program?

a. Yes

b. No

Thank you very much for your time!

Sincerely,

The Project Team
