# Supplementary figures and images for: Vaccine Hesitancy Phenomenon Evolution during Pregnancy over High-Risk Epidemiological Periods—“Repetitio Est Mater Studiorum”
Source: Vaccines (Basel). 2023 Jul 5;11(7):1207. doi: 10.3390/vaccines11071207 (PMC10384756; doi:10.3390/vaccines11071207)

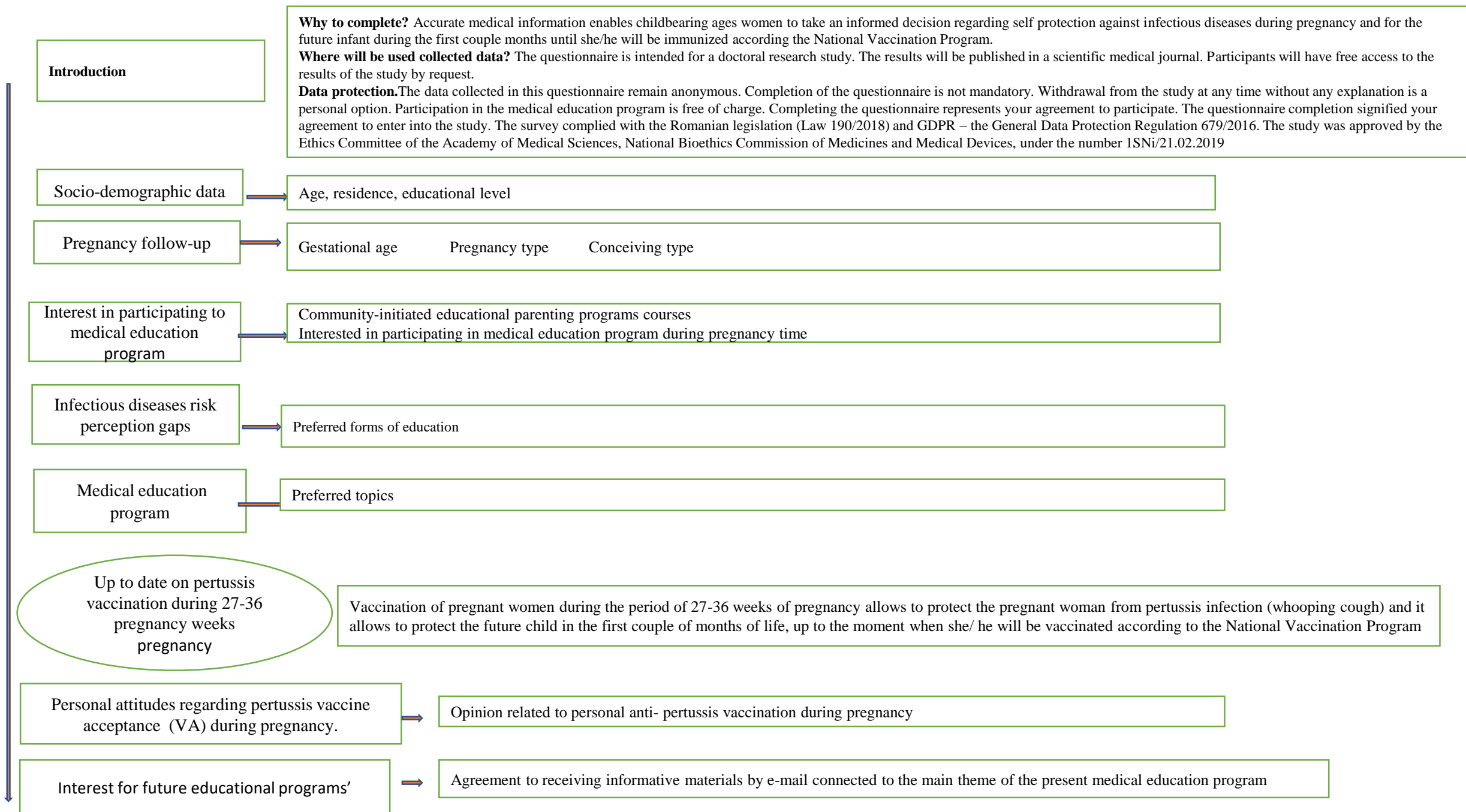

Supplement: Supplementary file 1 [file vaccines-11-01207-s001.zip › S2 Study FLow.pdf]
